# Bringing Homogeneous Iron Catalysts on the Heterogeneous Side: Solutions for Immobilization

**DOI:** 10.3390/molecules26092728

**Published:** 2021-05-06

**Authors:** Fabio Moccia, Luca Rigamonti, Alessandro Messori, Valerio Zanotti, Rita Mazzoni

**Affiliations:** 1 Dipartimento di Chimica Industriale “Toso Montanari”, Università degli Studi di Bologna, viale Risorgimento 4, 40136 Bologna, Italy; fabio.moccia@studio.unibo.it (F.M.); alessandro.messori6@unibo.it (A.M.); valerio.zanotti@unibo.it (V.Z.); 2 Dipartimento di Scienze Chimiche e Geologiche, Università degli Studi di Modena e Reggio Emilia, via G. Campi 103, 41125 Modena, Italy; luca.rigamonti@unimore.it

**Keywords:** iron catalysts, olefin polymerization, hydrogen transfer, immobilization, SILP, MCM-41, ionic liquid (IL), carbamate synthesis, alkene/alkane oxidation, chitosan

## Abstract

Noble metal catalysts currently dominate the landscape of chemical synthesis, but cheaper and less toxic derivatives are recently emerging as more sustainable solutions. Iron is among the possible alternative metals due to its biocompatibility and exceptional versatility. Nowadays, iron catalysts work essentially in homogeneous conditions, while heterogeneous catalysts would be better performing and more desirable systems for a broad industrial application. In this review, approaches for heterogenization of iron catalysts reported in the literature within the last two decades are summarized, and utility and critical points are discussed. The immobilization on silica of *bis*(arylimine)pyridyl iron complexes, good catalysts in the polymerization of olefins, is the first useful heterogeneous strategy described. Microporous molecular sieves also proved to be good iron catalyst carriers, able to provide confined geometries where olefin polymerization can occur. Same immobilizing supports (e.g., MCM-41 and MCM-48) are suitable for anchoring iron-based catalysts for styrene, cyclohexene and cyclohexane oxidation. Another excellent example is the anchoring to a Merrifield resin of an Fe^II^-anthranilic acid complex, active in the catalytic reaction of urea with alcohols and amines for the synthesis of carbamates and *N*-substituted ureas, respectively. A SILP (Supported Ionic Liquid Phase) catalytic system has been successfully employed for the heterogenization of a chemoselective iron catalyst active in aldehyde hydrogenation. Finally, Fe^III^ ions supported on polyvinylpyridine grafted chitosan made a useful heterogeneous catalytic system for C–H bond activation.

## 1. Introduction

The demand for increasingly efficient, economical, and environmentally friendly catalysts has prompted research to focus on transition metals alternative to the noble ones. Nowadays, catalysis is indeed dominated by platinum, palladium, rhodium, ruthenium, and iridium, which generally enjoy good selectivity. On the other hand, these metals are rare, expensive, and sometimes toxic. Iron has been emerging as one of the most promising alternatives by virtue of three great characteristics that distinguish it. First, after aluminum, iron is the most abundant metal in the Earth’s crust [1], which means wide availability and, therefore, low cost. Second, iron is biocompatible and plays a role in numerous biological processes [2]. This is particularly attractive for applications in the pharmaceutical, food and cosmetic industries. Furthermore, the environmental impact in relation to its wider and more widespread use is quite reassuring [3]. Finally, iron is able to catalyze a wide range of reactions, being second only to palladium in terms of versatility, thanks to the vast number of accessible oxidation states ranging from −2 to +6 [1]. The combination of these three aspects has promoted a real explosion in the last 15 years in the applications of iron compounds [4,5,6,7,8,9,10,11,12,13,14,15] in homogeneous catalysis [1], probably starting from what was defined “*a new iron age*” by C. Bolm in 2009 [16].

**Figure 1:**
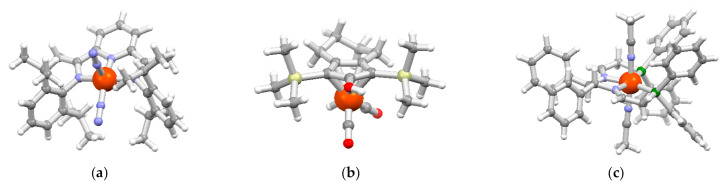
Crystal structures of (**a**) [Fe(*^i^*^Pr^PDI)(N_2_)_2_] (CCDC: 254793) [18], (**b**) the Knӧlker catalyst [Fe(η^5^-L)(CO)_2_(H)], L = 1,3-bis(trimethylsilyl)-4,5,6,7-tetrahydro-2*H*-inden-2-ol (CCDC: 114304) [19], and (**c**) [Fe(L’)(CH_3_CN)_2_](BF_4_)_2_ (L’ = (*R,R*)-*N*,*N’*-*bis*(2-(diphenylphosphino)ethylidene)-1,2-diphenylethylene-diamine (CCDC: 728131) [20], BF_4_ anions omitted for clarity. Structures reproduced with Mercury 4.3.1 [21], color code: Fe = orange, P = green, Si = yellow, O = red, N = blue, C = grey, H = white.

Iron has been historically used as a Lewis acid in aromatic electrophilic substitution reactions [17], but its use has recently received a strong development in new and very promising fields, such as, for example, the reduction reactions. In 2004, Chirik et al. were able to obtain the iron(0) complex [Fe(*^i^*^Pr^PDI)(N_2_)_2_] (*^i^*^Pr^PDI = 2,6-*bis*(2,6-diisopropylphenyl imino)pyridine) (Figure 1a) active in the hydrogenation of olefins under mild conditions [18]. In 1999, Knölker et al. instead synthesized the Knölker catalyst (Figure 1b) [19], efficient in the hydrogenation of aldehydes and ketones and, at the same time, capable of tolerating double and triple bonds [17]. These first pioneering publications were followed by other works. For example, Morris synthesized an iron catalyst bearing a diiminodiphosphino ligand (Figure 1c) capable of achieving an asymmetric hydrogenation of acetophenone with an enantioselectivity of 94% [20].

From a more peculiar perspective in green applications, molecular iron complexes have recently been found to be competitive in transformation for energy production, such as electro-catalyzed water oxidation [15,22,23,24,25]. On the other hand, plenty of room can be expected for their development in the underexplored world of iron complexes applied in biomass upgrading. Taking the transformation of bio-alcohols as an example [26,27], the advantages of higher conversions and selectivity (e.g., in alcohol homologation) demonstrate how homogeneous catalysis is more promising than the heterogeneous counterpart [28]. Nevertheless, this reaction still remains a prerogative of ruthenium catalysis [29,30,31,32].

The main drawback of the general challenge to move toward iron catalysis is that almost all the known iron catalysts nowadays are soluble species, and they are usually prefigured as homogeneous catalysts. This might involve several difficulties and burdens in industrial applications since, according to the classic definition by W. Ostwald [33,34], during the reaction the catalysts are in the same liquid phase, i.e., the solvent, as the reactants. This alone is already a good deterrent. Indeed, conventional solvents are commonly derived from petroleum and large volumes are generally required, resulting in a mix of unaffordability and low environmental compatibility that certainly depart significantly from the prerogatives of new sustainable processes. Other difficulties must also be underlined. The recovery and reuse of homogeneous catalytic species, when feasible, further burdens the already onerous processes of separation and purification, which are largely carried out through distillation columns. This can result in increased investment costs and energy demand. Furthermore, homogeneous catalysts might have low thermal stability and tend to decompose, definitively compromising their reuse. Homogeneous catalysis boasts two undeniable advantages, best selectivity and highest catalytic activity, but except for the cases where no heterogeneous catalysts are available and those where high selectivity is essential, it is always avoided on an industrial scale [35].

The affordability, versatility, and low environmental impact of iron-based catalysts bring them to our collective attention as a strategic solution in heterogeneous catalysis. The most common method generally used to achieve heterogenization is certainly immobilization on solid supports, where the catalytic species is bound to a solid matrix, generally silica, alumina or polymers, through the formation of ionic or covalent bonds [36,37,38,39,40,41,42]. These are followed by methods that provide supported liquid phases (aqueous or consisting of ionic liquids (ILs)) or liquid–liquid biphasic reactions. However, heterogenization processes can lead to a certain number of drawbacks, the first being the decreased activity due to the reduction of the contact surface. This is usually followed by a decline in selectivity, determined by the geometric modification of the active sites induced by the support. Furthermore, poor mechanical features of the substrates or chemical properties capable of unfavorably interfering during the reactions (non-innocent supports), are certainly deleterious factors. Finally, it is also important to pay attention to the degree of leaching of the catalyst in addition to its deactivation.

This review aims to investigate the current state of the art in the field of immobilization of iron catalysts, and to define the advances in heterogenization techniques to achieve the recycling and recovery of catalytic species. The numerous advantages offered by iron as an alternative to other transition metals and the criticalities that still prevent its widespread use will be discussed. The first examples, attributable to the early 2000s, employ immobilization on well-known and frequently used supports. This is the case of the *bis*(arylimino)pyridyl iron complexes supported on silica, used in the synthesis of polyolefins. Microporous molecular sieves (e.g., MCM-41 and MCM-48) are also described as supports for iron catalysts active both in the polymerization of olefins and in the oxidation of alkenes and alkanes, while iron compounds with anthranilic acid anchored on Merrifield resin are useful in the reaction of urea with alcohols and amines for the synthesis of carbamates and *N*-substituted ureas, respectively. Later, solutions characterized by an increasing green profile begin to emerge in relation both to the nature of the materials with which the catalytic systems have been made, and to the origin of these materials, and in relation to the processes used to obtain them. Therefore, a SILP-type (“supported ionic liquid phase”) catalytic system with iron complexes bearing pincer ligands has been employed in aldehyde hydrogenation, and immobilization of functional groups suitable for complexation of iron(III) on the bio-polymer chitosan is the last very recent example of a heterogeneous catalyst for C–H bond activation.

## 2. Heterogenization of Iron Catalysts for Olefin Polymerization

**Figure 2:**
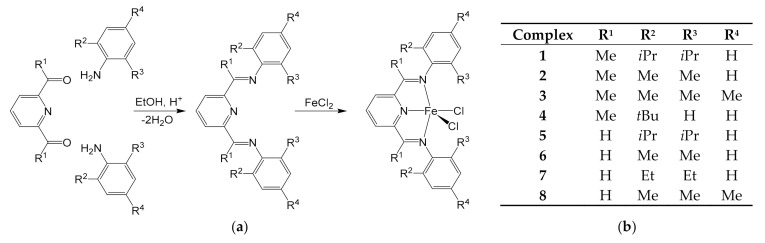
(**a**) The two-step synthesis of the iron pre-catalysts with *bis*(arylimino)pyridyl ligands for polymerization of olefins and (**b**) different combinations of R^1^, R^2^, R^3^ and R^4^ groups tested in complexes **1**–**8** [45].

At the end of the 1990s, particular attention was paid to the study of so-called “post-metallocene” catalysts to obtain increasingly productive and economical species for the synthesis of polyolefins. Among these, a category of iron complexes bearing *bis*(arylimino)pyridyl tridentate ligands proved to be particularly promising. Iron was indeed able to tolerate polar functional groups, allowing for the use of polar monomers in the synthesis of new copolymers. These properties are common to other “late transition metals” (cobalt, nickel) [43], but the advantage in the use of iron remained in its wider availability. Brookhart was the first to obtain *bis*(arylimino)pyridyl iron complexes and used them in the synthesis of polyolefins, exploiting an organo-aluminum co-catalyst [44]. The pre-catalyst was obtained through a two-step synthesis (Figure 2): condensation of 2,6-diacetylpyridine with two equivalents of a suitable aniline led to the ligands, followed by the complexation of iron, introduced as FeCl_2_. A first set of pre-catalysts was tested by Brookhart in the polymerization of ethylene [44], but only Gibson et al. later employed a broader spectrum of variously substituted pyridines and anilines to synthesize new ligands (Figure 2), with the aim of investigating the effects of substituents on the effectiveness of the catalyst [45]. The iron centers have a pseudo-square pyramidal structure in the pre-catalysts, where the three nitrogen atoms of the tridentate ligand and one chloride lay approximately in the same plane and the second chloride occupies the apical position. All the complexes turned out to be intensely colored (blue for ketimines and green for aldimines) and paramagnetic: the susceptibility measures were consistent with four unpaired electrons and high spin systems.

The complexes were then tested in the synthesis of polyethylene (PE) in solution, using isobutane as solvent and methylaluminoxane (MAO) as co-catalyst (results in Table 1) [45]. They confirmed that the whole family of catalysts was able to polymerize ethylene and, by virtue of the differences in terms of yield and average molecular weight of the polymer, it was possible to identify the key factors influencing the course of the synthesis. The chain propagation and transfer mechanisms that occurred during the ethylene polymerization were indeed investigated, and Gibson et al. performed a series of experiments through which they studied the effect of the individual parameters, like concentration of the MAO co-catalyst, reaction time and ethylene pressure [45].

Having defined the structure and reaction kinetics of these complexes, the need to heterogenize these catalysts started to be faced, since they have at least three major advantages:

(i) using MAO as co-catalyst, all the complexes had given good performances in the synthesis of PE, both in terms of yield and of average molecular weight, with excellent results in the case of the most encumbered complexes (catalysts **1** and **3** in Figure 2);
(ii) the synthesis of the *bis*(arylimino)pyridyl ligands took place with high yields. If we exclude the aldimine derivatives, which had, however, revealed less-interesting performances, all the ligands could be obtained from reagents already available on the market (2,6-diacetylpyridine, anilines and solvents). Finally, the complexation with iron took place in less than 2 h and it was almost quantitative;
(iii) the obtained PE turned out to be linear and of high density, so certainly interesting from an application point of view.

**Figure 3:**
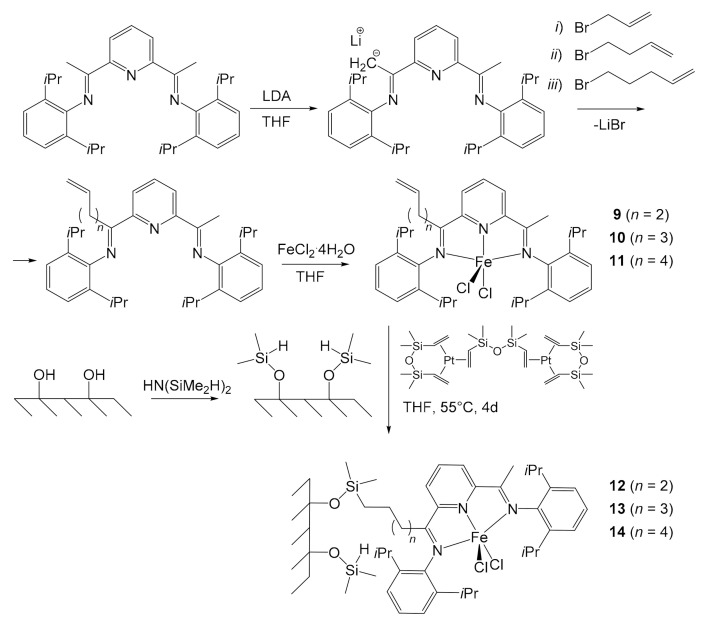
Herrmann’s functionalization of the iron pre-catalyst in three steps and immobilization on modified silica surface using the platinum-based Karstedt catalyst [47].

Herrmann et al. developed a first system to immobilize these iron pre-catalysts in 2002 [46], borrowing the idea from the techniques of immobilization of metallocenes, flourishing in those years [47,48]. The anchoring of 2,6-*bis*[1-((2,6-diisopropylphenyl) imino)ethyl]pyridine on silica was performed through the functionalization of the pre-catalyst (Figure 3). The pyridyl ligand was deprotonated with 1.05 equivalents of lithium diisopropylamide (LDA) in THF. Then, six equivalents of allyl bromide were added, and the mixture was heated under reflux. After extraction and purification, the desired product was obtained. Similarly, Herrmann reacted the deprotonated complex with 4-bromobut-1-ene and 5-bromopent-1-ene. The reaction time increased with increasing length of the bromides used, due to the steric hindrance. The obtained binders were dissolved again in THF and reacted with FeCl_2_⋅4H_2_O, yielding complexes **9**, **10** and **11**, with increasing length of the alkyl chain. The actual immobilization first entailed the modification of the silica surface by transforming the hydroxyl groups into -OSiMe_2_H ones through the reaction with 1,1,3,3-tetramethyldisilazane. The complexes were then fixed by hydrosilylation reaction between the alkenyl double bonds of the ligand and the Si−H groups of the silica, carried out using the Karstedt catalyst (Figure 3). The percentage of immobilized iron species ranged from 0.99 wt% to 1.15 wt%, depending on the alkenyl group of the complex.

Herrmann employed all pre-catalysts in the synthesis of PE before and after being immobilized to compare their performances. Complexes **9**, **10** and **11** were tested in solution using MMAO (modified MAO with the addition of long chain Al-alkyls) as co-catalyst (1000 equivalents with respect to iron), at a pressure of 2 bars of ethylene for 1 h. At 0 °C the catalysts showed an activity between 2.4 × 10^7^ and 1.07 × 10^8^ g PE/mol of Fe h bar. However, they deactivated very quickly as the temperature increased: they were already almost inactive at 20 °C after just 40 min. This behavior was explained by the tendency of the pre-catalysts to rapidly co-polymerize with ethylene through the alkenyl groups. Furthermore, the activity of the pre-catalysts decreased as the alkyl chain shortened. Conversely, the average PE molecular weight increased. Anchored pre-catalysts **12**, **13**, and **14**, loaded with 1.15, 1.10 and 0.99 wt% of iron, respectively, were subsequently tested under the same reaction conditions (amount of catalyst used: ~4 μmol Fe, 2.0 bar of ethene pressure, toluene as solvent, co-catalyst MMAO–Al/Fe = 1000, reaction time = 1 h). They all revealed good activity in the range of 10^3^–10^4^ kg of PE/(mol Fe h bar), albeit lower than their respective homogeneous catalysts of about an order of magnitude. On the other side, they all proved to be much more resistant than the corresponding pre-catalysts **9**, **10** and **11** to temperature increase, proving to be still active at 60 °C after 45 min, and this was certainly a great advantage. The slowdown in the deactivation of the catalyst was probably a direct consequence of the inability of the co-catalysts to copolymerize with ethylene. Unlike homogeneous complexes **9**–**11**, which produced polymers dominated by low *M*_W_, with the highest ones disappearing at polymerization temperatures > 20 °C, heterogeneous pre-catalysts **12**–**14** afforded a total *M*_W_ dominated by the high molecular fraction of the polymer of a mass of approximatively 10^5^–10^6^ g/mol (at a temperature of 0–40 °C). Only a small quantity of the polymer shows low mass (ca. 10^3^–10^4^ g/mol). In both cases, homo- and heterogeneous catalysis, *M*_W_’s decrease with the increase of temperature. The highest *M*_W_’s were obtained with the pre-catalyst **12** bearing a short alkenyl moiety. Finally, no fouling of the reactor was observed during the PE synthesis, unlike with homogeneous pre-catalysts. Herrmann did achieve an important result, but the immobilization process was long and laborious, and the quantities of iron immobilized were relatively low. In addition, anchoring involved the use of a platinum-based catalyst, notoriously expensive although very selective for this step [49].

**Figure 4:**
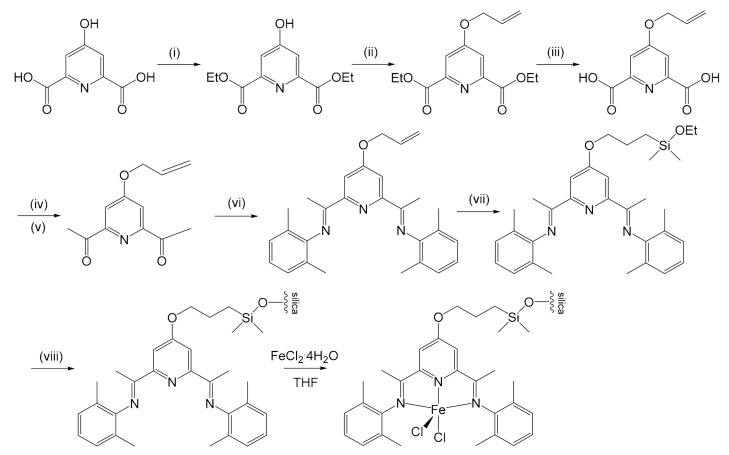
Kim’s immobilization strategy over silica for iron pre-catalyst [50]. Conditions: (i) EtOH, concentrated H_2_SO_4_, 90 °C; (ii) K_2_CO_3_, allyl bromide, acetone, reflux; (iii) 5 eq L^−1^ NaOH, THF, 50 °C; (iv) SOCl_2_, DMF, 90 °C; (v) CuI, MeLi, Et_2_O, THF, −78 °C; (vi) 2,6-dimethylaniline, EtOH, AcOH, reflux; (vii) (CH_3_)_2_SiHCl, H_2_PtCl_6_⋅6H_2_O (catalyst), CH_2_Cl_2_, EtOH:Et_3_N 1:1; reflux; (viii) silica gel, toluene, 120 °C.

Kim et al. proposed a new method for the immobilization of the same complex on silica in 2003 [50]. The result was interesting because the activity of the pre-catalyst, which loaded 0.199 mmol of Fe/g, in the synthesis of PE turned out to be 10 times greater (4.87 × 10^7^ g PE/mol Fe h bar against 4.53 × 10^6^ g PE/mol Fe h bar). However, the anchoring method developed was not much easier (Figure 4): an esterification was performed from 4-hydroxypyridine-2,6-dicarboxylic acid in ethanol to obtain the diethyl ester, in the presence of sulfuric acid. The product was alkylated in acetone with allyl bromide in the presence of K_2_CO_3_. The esters were then hydrolyzed with a 5 mol L^−1^ NaOH aqueous solution in THF to obtain the carboxylic acid, followed by the formation of acyl chloride with SOCl_2_ and then its acylation by reaction with methyllithium, catalyzed by CuI. The *bis*-iminopyridine could therefore be prepared through a condensation reaction between 4-(allyloxy)-2,6-diacetylpyridine and 2,6-dimethylaniline. Hydrosilylation was then conducted with chlorodimethylsilane using H_2_PtCl_6_⋅6H_2_O as catalyst. The last two steps involved the formation of the Si–O–Si covalent bond that fixed the ligand on silica, and the formation of the complex by reaction with FeCl_2_⋅4H_2_O. Supported catalysts showed lower activity with respect to the homogeneous counterpart, probably due to reduced number of active sites. On the other hand, it allows the obtaining of a PE with higher *M*_W_ that is not run time dependent: *M*_V_ (ca. 1.6 × 10^5^) after either 3 min or 1 h reaction time. As for Herrmann’s previously described case, *M*_W_ of the polymer decreased while increasing the temperature for both supported and unsupported catalysts. Thus, since the activation energy for the termination is somewhat higher than that for propagation, the *M*_W_ can be controlled by the temperature. Although offering better catalytic performances, some critical issues in the synthetic process remained: hydrosilylation still required a platinum catalyst, and the extensive use of halides certainly did not make the process less impactful from an environmental point of view.

**Figure 5:**
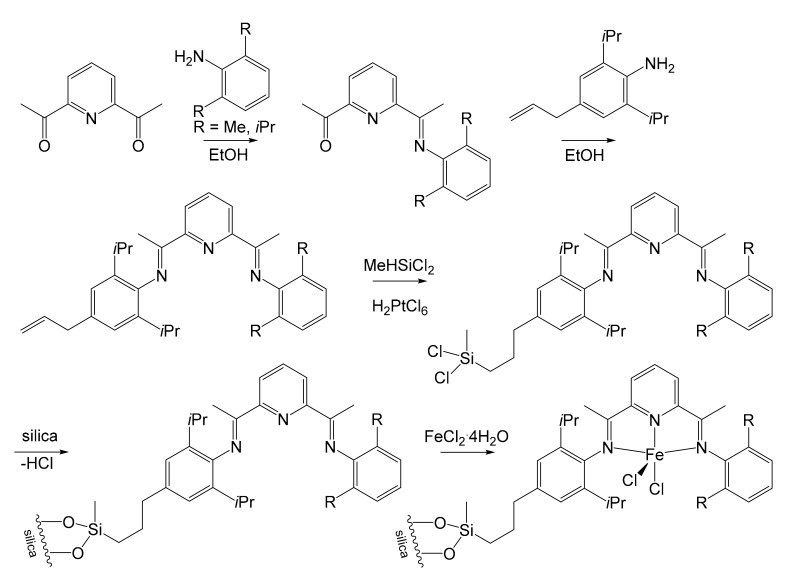
Li’s immobilization strategy over silica for iron pre-catalyst [51].

A final proposal to immobilize *bis*(arylimino)pyridyl iron complexes on silica was put forward by Li et al. in 2005 [51]. Its main merit was the significant simplification of its implementation, even if not being truly innovative. The immobilization step continued to be based on a platinum-catalyzed hydrosilylation reaction followed by the reaction with the silanol groups of the silica. However, the alkenyl double bond was already introduced into the complex through the condensation of anilines with pyridine, allowing for a double advantage in terms of yield and use of reagents (Figure 5). These pre-catalysts immobilize a higher amount of iron (between 2.69 wt% and 3.70 wt%) if compared with Kim’s and Herrmann’s supports, because of the easy reaction between Si–Cl and Si–OH. Anyway, this strategy proved to be less performing from an activity point of view (≈1 × 10^6^ g PE/mol Fe h bar at 25 °C) than those previously developed by Herrmann and Kim, but could be more easily synthesized. Even in this case, much higher *M*_W_’s and lower catalytic activity were registered for the supported catalyst. Unimodal mass weight distribution was observed. This can be attributed to the steric surrounding due to silica, which hinders β-H transfer reaction and chain transfer toward organic aluminum to some extent.

**Figure 6:**
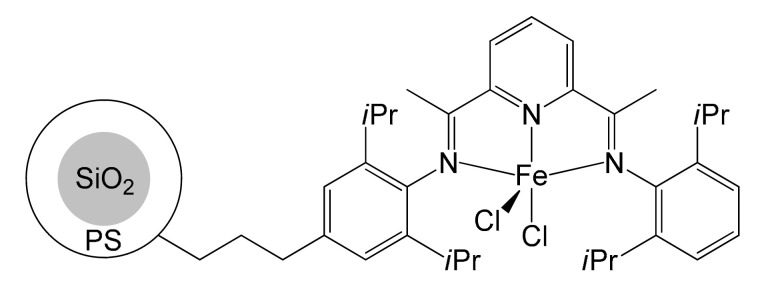
Immobilization of iron pre-catalyst performed by Jin and Liu through copolymerization with polystyrene (PS) [52].

Simultaneous to the attempts of immobilization on silica, a new path was explored by Jin and Liu, who managed to immobilize the iron complex by co-polymerization with polystyrene (PS) on silica spheres [52]. The pre-catalyst was synthesized by employing already functionalized anilines, in a manner similar to what Li had previously done. Subsequently, spheres of silica, styrene, a small amount of divinylbenzene and the iron pre-catalyst were introduced in a reactor containing toluene as solvent. The addition of AIBN (azobisisobutyronitrile) as radical initiator triggered the polymerization reaction in solution where the pre-catalyst copolymerized with PS deposited on the silica spheres (Figure 6).

The key to obtaining good support lay in the right dosage of divinylbenzene. An excess of this, in fact, led to the synthesis of a highly cross-linked insoluble PS, which immediately precipitated with no uniform deposition on silica. On the other hand, too small a quantity of divinylbenzene led to the obtaining of a very fragile copolymer that crumbled easily once the solvent was removed due to the “swelling” phenomenon. Another important factor was the thermal control of the reaction, since high temperatures seemed to favor homo-polymerization of the iron complex, avoiding co-polymerization with styrene. The best results were obtained between 79 and 81 °C, with a crosslinking degree of 4.07%, calculated with respect to the initial reagents. This allowed them to fully exploit the advantage offered by silica, considerably increasing the immobilization surface by minimizing the amount of complex used. The final material had a rather low quantity of immobilized iron equal to 0.14 wt%. However, it revealed an extraordinary activity in the synthesis of PE, equal to 3.18 × 10^6^ g PE/mol Fe h at 60 °C and 2.96 atm of ethylene. The PE obtained was linear with high molecular weight (*M*_V_ = up to 94 × 10^4^ g/mol at 51 °C) and with a rather regular spherical morphology. Furthermore, the synthetic process and the heterogenization of the iron complex were simpler to carry out compared to the methods previously described. All these factors can lead to designating the last strategy as the best immobilization technique among those illustrated.

In the meantime, molecular sieves MCM-41 were also investigated by Schuchardt et al. in 2004, as support for similar 2,6-*bis*(imino)pyridyl ligands to exploit the high surface area and well defined pore diameter [53]. The heterogeneous catalysts were prepared by simply stirring 1.0 g of MCM-41 with 45 or 38 μmol of the molecular complexes of the type reported in Figure 2 (**1** and the congener **I** with R^2^ = R^3^ = Me and R^4^ = Br as substituents) in 10 mL of toluene for 4 h, without the need for further heterogenizing agents. Washing with toluene to remove the residual non-encapsulated complexes revealed an almost quantitative occlusion of the iron complexes in MCM-41 (43 μmol/g for **1-**MCM41 and 35.8 μmol/g for **I-**MCM-41), promoted by the shape of the pores, which allowed for a very good absorption without the need of MAO as fixing agent. MAO was then added to activate the catalysts. **I-**MCM-41 showed more promising results than **1**-MCM-41, even if both complexes were less active than under homogeneous conditions. Melting point and molecular weight of the obtained PE increased, with the latter up to 10 times after the catalyst immobilization. This behavior shows that the support is efficient in reducing chain transfer reactions, likely due to large steric hindrance, around the active centers given by the channel walls. The effect of ethylene pressure on molecular weight was also screened. Although the increasing of molar mass requires the use of high pressure, the pressure required remained lower than that needed for iron complexes supported on silica [46], representing a step forward for these hybrid materials toward industrial PE production.

**Figure 7:**
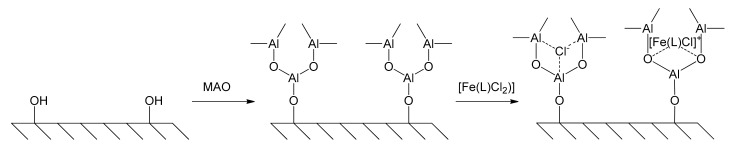
Immobilization of 2,6-*bis*[1-(2,6-diisopropylphenylimino)ethyl]pyridyl dichloro iron(II) on MCM-41 [54].

Hu and co-workers published in 2005 about the supporting of the catalyst 2,6-*bis*[1-(2,6-diisopropylphenylimino)ethyl]pyridyl dichloro iron(II), [Fe(L)Cl_2_], on MCM-41 [54]. Molecular sieves, previously treated with MAO to modify the mesoporous MCM-41 by reaction with the silanol groups lined along their internal walls, were then allowed to react in toluene with the iron complex, which can be tethered on MCM-41 by the bridge of MAO (Figure 7).

The final heterogeneous catalyst has an iron loading of 0.20 wt%. Although the activity of the immobilized species is still lower than that of the homogeneous counterpart, good catalytic performances, around 3 × 10^6^ g mol^−1^ h^−1^ atm^−1^, are registered in comparison to the silica systems previously described. The increasing molecular weight with respect to the homogeneous catalyst confirms the role of molecular sieves in suppression of the β-hydrogen transfer mechanism.

## 3. Immobilization of Iron Catalysts on Molecular Sieves for Oxidation Reactions

**Figure 8:**
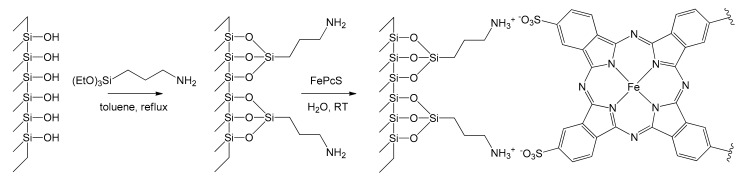
Immobilization of FePcS on the inner surface of functionalized mesoporous MCM-48 and MCM-41 silica substrates [55].

Remaining in the field of immobilization of iron catalysts on molecular sieves, Safari et al. exploited this approach in 2008 toward the heterogenization of iron tetrasulfophthalocyanine (FePcS) [55]. This complex was anchored on the surface of functionalized MCM-48 and MCM-41 silica substrates by means of chemical bonding to aminosilane groups. Mesoporous silicas were grafted with propylamine groups by treating 1 g of MCM-41 or MCM-48 with 1.6 g of (EtO)_3_SiCH_2_CH_2_CH_2_NH_2_ in refluxing toluene for 2 h. Then, 40 mg of FePcS in aqueous solution was reacted with 1 g of the functionalized mesoporous silica obtaining a precipitate, which was filtered and washed with water (Figure 8).

Leaching experiments showed very good stability of the supported systems in water, making them suitable for heterogeneous catalysis. Preliminary studies on the catalytic activities of FePcS/NH_2_-MCM-48 (FePcS = 16 wt%) and FePcS/NH_2_-MCM-41 (FePcS = 8 wt%) were performed in the oxidation of styrene (catalyst loading: 4%) employing *tert*-butyl peroxide as oxidant in a mixture of methanol and water at room temperature. Heterogeneous catalysts demonstrated a superior durability and activity under milder conditions compared to the homogeneous catalyst. Furthermore, FePcS leads to the formation of undesirable benzoic acid, while the selectivity is toward benzaldehyde in the supported catalysts (Table 2).

Interestingly, FePcS/NH_2_-MCM-48 performs better than FePcS/NH_2_-MCM-41. This can be attributed to the three-dimensional pore structure of FePcS/NH_2_-MCM-48, which provides an easier access to guest molecules. On the other hand, higher surface area and larger pore size, which favor the adsorption of styrene, can also be taken into consideration to explain the higher activity. Although no leaching of active species into the solution could be detected and stability studies under catalytic conditions show a higher durability of the supported catalysts, this latter had lower activity on the second run, preventing the actual recyclability of the heterogeneous systems.

Dias et al. anchored tetraphenylporphyrin (TPP) complexes of various metals from the first transition series in MCM-41 [56]. Concerning the iron complex Fe(TPP)Cl, immobilization was achieved by stirring the support MCM-41 in a CH_2_Cl_2_ solution of the metalloporphyrin for 48 h and then under reflux for 1 h, allowing the encapsulation of Fe(TPP)Cl into the pores of the support. Soxhlet technique was employed to remove the weakly adsorbed species on the mesoporous surface. FeTPP-MCM-41 was obtained with an amount of metal anchored up to 2.06 wt%, and the supported system showed an increased stability of the complex from 256 to 350 °C after immobilization. This can be related to the stabilization of the complex after the interaction between the metal and the silanol OH groups of MCM-41. Even in this case no leaching was observed. The anchored catalyst was tested in the oxidation of cyclohexene with hydrogen peroxide, showing better results in terms of conversion compared to cobalt and manganese congeners, with this latter having a better TON.

**Figure 9:**
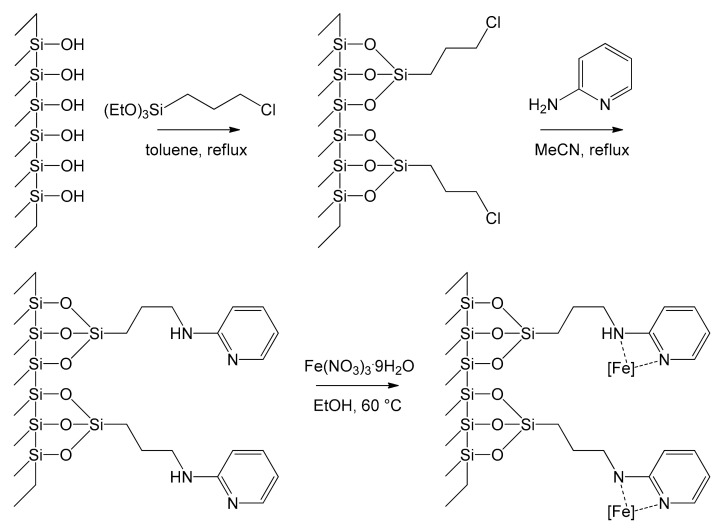
The stepwise preparation of MCM-Py-Fe(III) from MCM-41, [Fe] = anchored complex [57].

Another work principally devoted to synthesis and characterization was published by Adam et al. in 2013 [57]. They grafted 2-aminopyridinyl iron(III) complexes to chlorosilane-modified MCM-41, leading to the formation of the novel immobilized system MCM-Py-Fe(III). The first step was the functionalization of MCM-41 pores with alkyl chloride groups by refluxing MCM-41 with 3-chloropropyltrietoxysilane (CPTES) in toluene for 24 h to produce MCM-PrCl. This latter was refluxed with aminopyridine in acetonitrile for 24 h to produce MCM-Py. Once the ligand was covalently linked to the support, it was refluxed with Fe(NO_3_)_3_⋅9H_2_O in ethanol at 60 °C for a further 24 h, leading to the hybrid material MCM-Py-Fe(III) (Figure 9). This latter is a nice example of covalently bonded complexes toward stable hybrid materials. The work nicely describes several characterizations being the first in exploiting ^15^N solid state MAS-NMR for the characterization of hybrid mesoporous MCM-41 materials.

Several iron(III) complexes have been recently synthesized and used as biomimetic metalloenzymes for heterogeneous hybrid catalytic systems for alkane (e.g., cyclohexane) oxidation. Examples are Fe(salen) complexes encapsulated into zeolite Y [58], iron(III) porphyrins intercalated into layered double hydroxides [59,60,61], occluded in zeolite X [62], immobilized on silica surface and encapsulated in silica matrix [63], supported on montmorillonite [64], kaolinite [65] or onto in situ obtained zinc oxide [66]. Covalently grafted systems have also been developed with Fe(III) pyridine-carboxylate complexes on kaolinite [67]. The non-heme iron complexes [Fe(en)_2_Cl_2_]Cl, [Fe(bpy)_2_Cl_2_]Cl, [Fe(salen)Cl], [Fe(TPP)Cl] and [Fe(TMC)Cl], where en, bpy, salen, TPP and TMC refer to ethylenediamine, 2,2-bipyridine, *N,N*-*bis*(salicylidene)ethylenediamine, *meso*-tetraphenylporphyrin and 5,7,12,14-tetramethyl-1,4,8,11-tetraazacyclotetradeca- 4,6,11,13-tetraene, respectively, were immobilized within nanoreactors of Al-MCM-41 by Farzaneh’s group [68]. Conditions for heterogenization are similar to those reported above for MCM-41 mesoporous materials, namely occlusion or formation of covalent bonds between the ligand and the support, subsequently followed by coordination reaction with an iron(III) precursor. All the mentioned heterogeneous systems were active in the oxidation of cyclohexane and other alkanes. Although the majority of complexes for oxidation of cyclohexane are based on porphyrins, good performances have been more recently found by Farzaneh et al. with the biomimetic non-heme complex [Fe(L)_2_OH(H_2_O)_3_] (L = pyridoxinato) immobilized within the nanoreactor Al-MCM-41 [69]. With a 3.34 wt% of supported iron species, the heterogenized catalyst works better both in conversion (86%) and in selectivity (88% cyclohexanone vs. 12% cyclohexanol) compared to the homogeneous counterpart (conversion = 47%, selectivity: 60% cyclohexanone vs. 40% cyclohexanol). This latter catalyst [Fe(L)_2_OH(H_2_O)_3_]/Al-MCM-41 was recovered and recycled three times, and the amount of iron was maintained with a minor decrease in conversion.

## 4. Heterogenization of Iron Catalysts on Merrifield Resin

**Figure 10:**
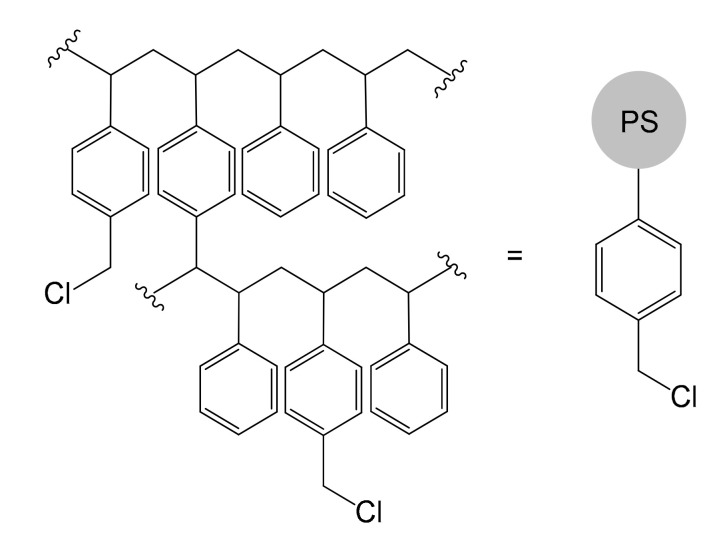
Structure of the functionalized polymeric resin realized by Merrifield in 1963 [70].

The work published by Robert Bruce Merrifield in 1963 entitled “Solid Phase Peptide Synthesis. I. The Synthesis of a Tetrapeptide” [70] constitutes a milestone in the context of the synthesis of solid supports and, more generally, in the heterogenization of reactions. Merrifield had the merit of devising a versatile system to overcome a very difficult problem at the time: the laboriousness of the polypeptide synthetic process, which required a tedious process of orthogonal protection and deprotection of the various functional groups. He had the intuition to fix the first amino acid to a solid support, thus managing to develop a mechanized process, which allowed him to synthesize even very long chains in times unimaginable until then. For the enormous contribution made to the synthesis of polypeptides, R. B. Merrifield won the Nobel Prize in 1984 and, probably unconsciously, he also contributed to the entire panorama of organic synthesis [71]. In fact, the Merrifield resin, a cross-linked PS functionalized with chloromethyl groups (Figure 10), has undoubtedly become one of the most widespread and used support systems, prefiguring itself, after decades of use, as one of the most reliable materials for making an immobilization.

**Figure 11:**
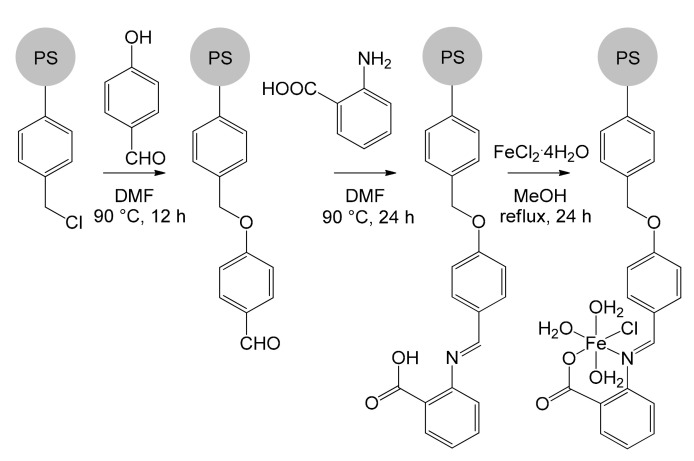
Synthesis of the catalyst [Fe^II^(Antra-Merf)] immobilized over a PS Merrifield resin [72].

Recently, Basu’s group immobilized an iron catalyst by exploiting a Merrifield resin functionalized with anthranilic acid (Figure 11) [72]. The resulting [Fe^II^(Antra-Merf)] catalyst constitutes a green, simple and low-cost solution for the synthesis of carbamates and *N*-substituted ureas. The catalyst immobilization process took place in four steps. Initially, 4-hydroxybenzaldehyde was added dropwise to a suspension of the Merrifield resin in DMF. The hydroxyl group performed a nucleophilic substitution on the chloromethyl fragment of the resin. The product was isolated by filtration after having heated the mixture at 90 °C for 12 h. Subsequently, anthranilic acid was added, leading to the formation of an imine bond and the product, by virtue of the anchorage to the resin, could be again isolated by simple filtration. Finally, the last step involved the addition of iron(II) chloride under nitrogen atmosphere in methanol, obtaining the immobilized catalytic species.

The catalyst thus obtained was first used in the synthesis of carbamates. The interest in carbamates resides in their transversal use in several areas, which range from insecticides to cosmetic products [73,74]. In addition, they play a role in the production of countless artifacts such as wood preservatives and stabilizing agents [75]. Finally, they are widely used in pharmacology and in chemical synthesis as protection of –NH_2_ groups [76]. The main synthetic route for carbamates involves the fixation of CO_2_ with amines and a series of various substrates, consisting mainly of halides and alcohols [77,78,79,80]. This route involves a series of technical difficulties: production of toxic or polluting by-products, need for high pressures of carbon dioxide, use of homogeneous catalysts.

**Figure 12:**
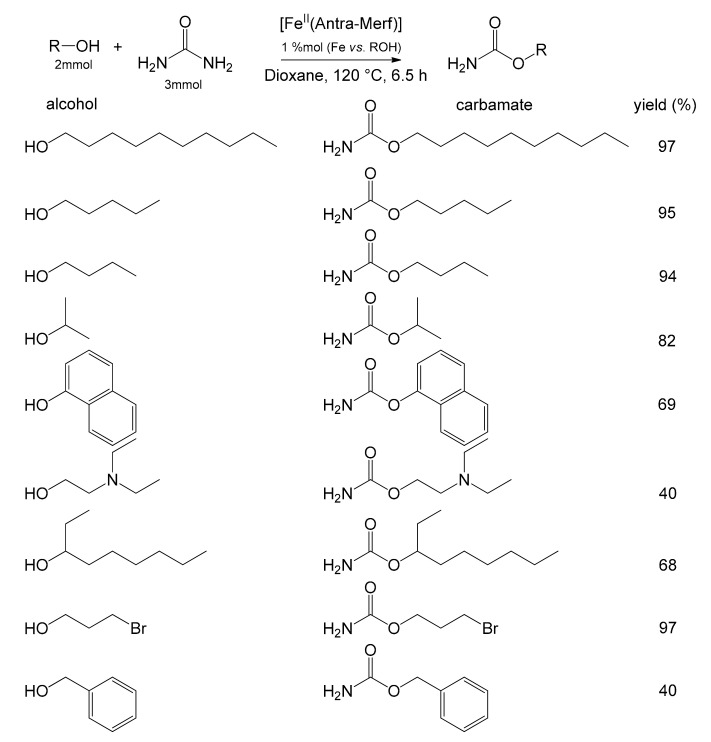
Reaction between urea and different alcohols with the catalyst [Fe^II^(Antra-Merf)] yielding carbamates [72].

The solution devised by Basu et al., using the anchored iron complex as an electron acceptor, turned out to be a simple and low-impacting synthesis of carbamates by direct reaction of urea with alcohols (Figure 12). The authors referred to the reaction between urea and benzyl alcohol to determine the optimal reaction conditions for the heterogeneous system [72]. They determined that the best yield was obtained using 1,4-dioxane as solvent and that, in general, all non-polar solvents favored the reaction. Conversely, all polar solvents inhibited the reaction. The increase in the catalyst amount affected the yield up to a plateau of about 97%. The best results were obtained by carrying out the reaction for 6.5 h at 120 °C. Finally, a comparison was performed with the same catalyst in the homogeneous phase. Surprisingly, it showed a lower reaction yield than that of the immobilized species, probably due to difficulties in product separation and purification. The substrate’s scope was then investigated by adopting the optimized parameters, highlighting the extreme versatility of the catalyst (Figure 12).

**Figure 13:**
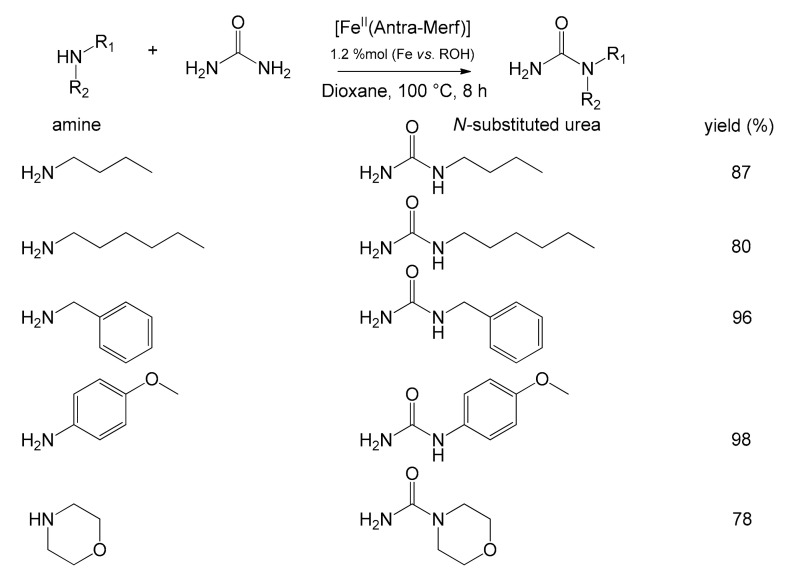
Reaction scheme between urea and differently substituted amines with the catalyst [Fe^II^(Antra-Merf)] yielding *N*-substituted ureas [72].

In the same work, the authors also studied the efficacy of the [Fe^II^(Antra-Merf)] catalyst in the synthesis of *N*-substituted ureas (Figure 13), widely requested by the market as they are used in the synthesis of ILs [81], pesticides and herbicides [82].

*N*-substituted ureas can be synthesized through a nucleophilic substitution reaction of an amine on urea, and several systems employing homogeneous catalysts have been devised [83,84]. Unfortunately, they are difficult to apply on an industrial scale due to the difficulties in the processes of separation and reuse of catalytic species [72]. The catalyst [Fe^II^(Antra-Merf)] proposed has shown excellent catalytic capabilities in this reaction, even if limited to the use of primary amines or aliphatic amines without electron-withdrawing groups (Figure 13).

**Figure 14:**
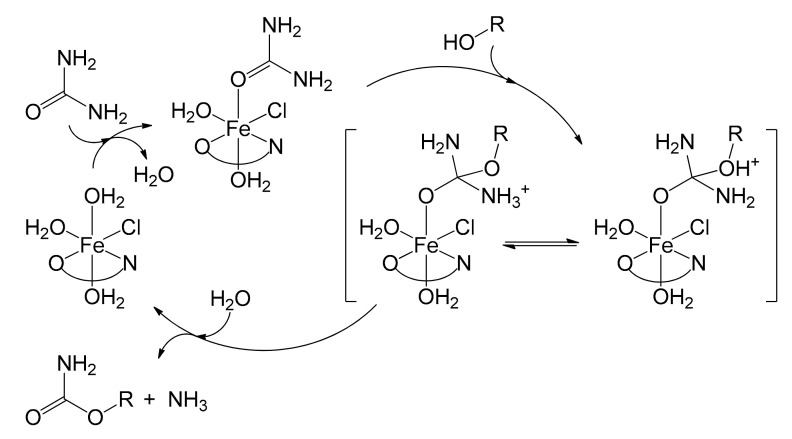
Proposed reaction mechanism between urea and alcohols in the presence of the catalyst [Fe^II^(Antra-Merf)] (Antra-Merf = O^N) [72].

The published study [72] proposed a plausible mechanism, illustrated in Figure 14 for alcohols. In the first step, urea was activated by the immobilized iron complex leading to the formation of a species where urea was coordinated to iron. This accentuated the electrophilic character of the carbonyl group, allowing the alcohol to perform a nucleophilic addition. The resulting intermediate underwent an intramolecular proton transfer from the alcohol to one of the amino groups. At this point, ammonia and carbamate were released by decomposition with regeneration of the catalytic species. The catalyst, as described, appeared to be an excellent alternative to the solutions previously adopted, especially considering the very low iron leaching after its consecutive reuse six times (0.028 ppm). This further confirms the effectiveness of the heterogenization method adopted.

## 5. Heterogenization through Catalytic SILP Systems

**Figure 15:**
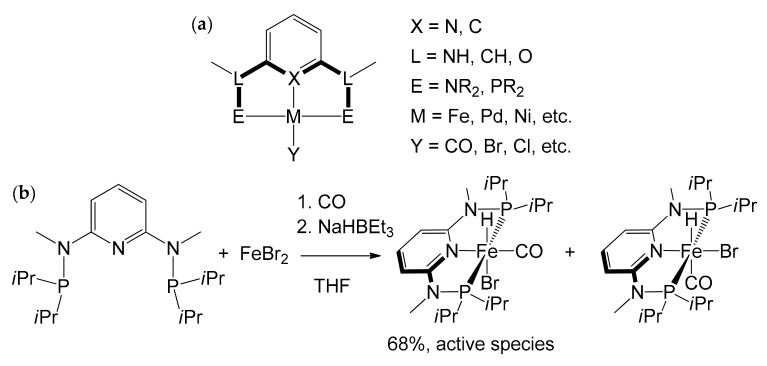
(**a**) General formula for metal complexes with ‘pincer’ tridentate ligands; (**b**) synthesis of the complex [Fe(PNP-Me-*i*Pr)(CO)(H)(Br)] performed by Kirchner et al. as mixture of isomers, where the active species in catalytic hydrogenation reaction of C=O double bonds is the one with H in *trans* to Br [92].

Already in the mid-1970s of the last century [85,86], a new type of complex, bearing “pincer” ligands, had emerged strongly on the panorama of homogeneous catalysis. These peculiar complexes are composed by a tridentate ligand, which establishes a meridional coordination with the metal center. Although there is no stringent definition that delimits its composition, the tridentate ligand generally consists of a central aromatic ring bearing an atom that can establish a σ-donation, and two substituents on the two adjacent positions whose ends also coordinate the metal (Figure 15a). The components of each portion are extremely variable, but, generally, the central atom (**X**) is made up of carbon or nitrogen. The NH, O or CH_2_ groups generally act as “linkers” (**L**) with the coordinating ends (**E**) that, on the other hand, are typically made up of amine –NR_2_ or phosphine –PR_2_ groups. The pincer ligands are indicated with an abbreviation containing the symbols of the three coordinating atoms (PNP, NNN etc.), where the second symbol always identifies the central coordinating atom. By virtue of such a strong interaction, the resulting complexes are highly thermally stable and have revealed interesting catalytic properties in various applications: hydrogenation reactions of olefins, amides, nitriles, esters and dehydrogenation, cross-coupling, hydroboration, and hydrosilylation [87,88,89,90,91].

In 2014 Kirchner et al. synthesized a catalyst for the hydrogenation reaction of C=O double bonds, based on the complex [Fe(PNP-Me-*i*Pr)(CO)(H)(Br)] [92]. It was obtained by treatment of anhydrous FeBr_2_ with 1 equivalent of PNP-Me-*i*Pr pincer ligand in THF in the presence of CO, and by subsequent addition of NaHBEt_3_ (Figure 15b). The reaction led to the formation of two isomers; the one with H in *trans* to Br, the active species in the hydrogenation catalysis, can be isolated with a yield of 68%. The discovery proved to be of considerable interest, as the catalyst is active and chemoselective for the hydrogenation of aldehydes in the presence of ketones, esters, and C=C double bonds. The pre-catalyst [Fe(PNP-Me-*i*Pr)(CO)(H)(Br)] was initially tested on some simple aldehydes as substrates, like benzaldehyde and 2-pyridinecarbaldehyde (Table 3), leading to almost quantitative yields in all cases (99%) and TOF ≈ 120 h^−1^. Two important factors emerged while identifying the optimal reaction conditions: first, the reaction did not work if carried out in an aprotic solvent; second, the reaction did not proceed if a strong base was not present in the reaction environment.

**Figure 16:**
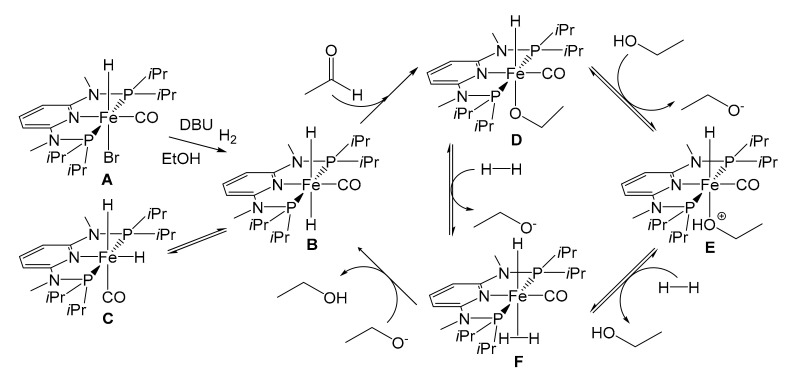
Hydrogenation mechanism of acetaldehyde proposed by Kirchner et al. (DBU = 1,5-diazabiciclo(5.4.0)undec-7-ene) [93].

In 2016 [93], taking the hydrogenation of acetaldehyde as a model, Kirchner investigated a plausible reaction mechanism capable of explaining all the experimental findings with spectroscopic studies and DFT calculations (Figure 16). The substrates tested were further expanded (Table 3) [93] and it was finally possible to observe that the catalyst acts selectively on aldehyde groups in the presence of double C=C bonds and α-β-unsaturated systems.

In order to fill the gap between a promising system and an actual possibility for industrial application, the next step was to immobilize the pre-catalyst [94]. In view of exploiting a solution with little impact from an environmental point of view but still functional to the practical needs of catalyst recycling, the authors resorted to a SILP catalytic system. They consist of three components: a porous solid support, an IL and the catalytic (or pre-catalytic) species. The process requires impregnation of the solid support with the IL already dissolving the catalyst, producing a supported liquid phase. A key role is therefore assumed by ILs with their low melting points (below 100 °C) and very low vapor pressures. These last features make ILs exceptional solvents, combined with high solvating capacity. The real peculiarity of ILs is the control of their chemical-physical properties (viscosity, melting point, miscibility in water, density, etc.) through the combination of suitable ionic species, allowing a real design of the solvent. Organic cations such as tetraalkylphosphonium, tetraalkylammonium and *N*-alkyl- pyridinium are generally used, and *N,N′*-dialkylimidazolium cations are much more recurrent in SILP catalysis. Anions are more frequently inorganic species such as Cl^−^, Br^−^, PF_6_^−^, CF_3_SO_3_^−^, (CF_3_SO_2_)_2_N^−^ [95].

It is important to underline that ILs were initially used in liquid/liquid biphasic catalysis, with the aim of maintaining the high selectivity and activity that distinguish homogeneous species while being able to exploit the immiscibility of the two phases to recover the catalyst [96]. Despite expectations, the implementation of the process at an industrial level was immediately impracticable, due to the following reasons:

(i) the large quantities of IL compromise the affordability of the process, both for the intrinsic cost of the solvent and for the costs of its disposal and/or recycling;
(ii) separation of the two phases, the polar and the apolar ones, requires the use of organic solvents, increasing costs and lowering the environmental sustainability;
(iii) the typically high viscosity of ILs results in problems related to the transfer of matter, confining the reaction to a thin layer near the interface between the two phases. This leads to substantially inactive catalytic species dispersed in the ILs.

These drawbacks can be overcome thanks to the introduction of a solid support. This allows considerable reduction of the consumption of IL, increase of the exchange surface by acting on the size of the particles and the size of the pores of the support, solving of the problems related to viscosity, and clear simplification of the separation process thanks to the introduction of a solid phase. Usually, the support consists of silica with a high surface area (300–500 m^2^ g^−1^) and, only rarely, alumina by virtue of its greater stability at high pH values. Polymeric supports have also been explored [97,98].

**Figure 17:**
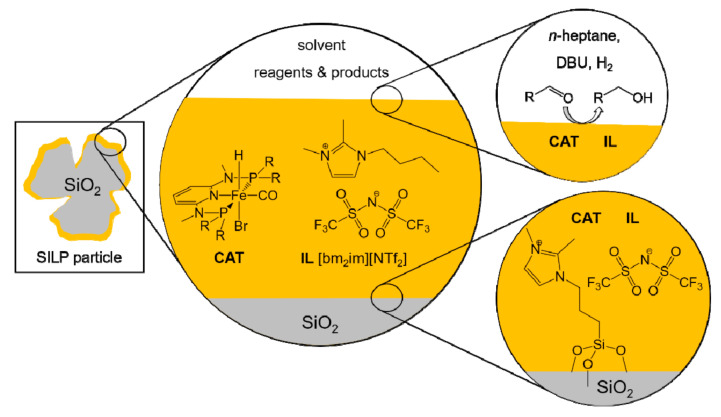
Structure of the SILP catalytic system exploited by Kirchner et al. with [bm_2_im][NTf_2_] as **IL** anchored on silica particles and [Fe(PNP-Me-*i*Pr)(CO)(H)(Br)] as pre-catalyst (**CAT**) [94].

Kirchner’s SILP catalytic system was thus composed by the pre-catalyst [Fe(PNP-Me-*i*Pr)(CO)(H)(Br)], dissolved in 1-butyl-2,3-dimethylimidazolium *bis*(trifluoromethylsulfonyl)imide ([bm_2_im][NTf_2_]) (Figure 17) [94]. Silica gel was chosen as the support, previously treated at 400 °C to reduce the OH groups and then functionalized with 1,2-dimethyl-3-(3-trimethoxysilylpropyl)imidazolium chloride. Treatment with lithium *bis*(trifluoromethylsulfonyl)imide (Li[NTf_2_]) led to the exchange of the anionic species with the production, even if only partial, of a real immobilization of the IL through covalent bonds. This expedient allowed them to strongly diminish the leaching phenomena of both the IL and the catalyst. The contemporary neutralization of the surface –OH groups of the silica is fundamental since the pre-catalytic species **A** and **B** in Figure 16 are basic and the presence of acidic hydrogen atoms in the reaction environment would lead to a deactivation of the catalytic species. The evaluation of the pre-catalyst loading proceeded with the impregnation (Figure 17), with variable percentage by weight of homogeneous catalyst dissolved in the IL: SILP10 (10 wt%), SILP20 (20 wt%), SILP30 (30 wt%), SILP40 (40 wt%).

The four systems were tested in the hydrogenation of 4-fluorobenzaldehyde to 4-fluorobenzyl alcohol, in the presence of DBU (5 mol%) as base and *n*-heptane as solvent at 25 °C [94]. SILP20 emerged as the most efficient species to catalyze the reaction (Table 4), producing a yield of over 99% in 17 min with 10 bars of hydrogen (TON = 200; TOF = 706 h^−1^). The authors also conducted the reaction in the homogeneous phase (TON = 200 TOF = 2000 h^−1^) and in the biphasic system (TON = 200; TOF = 1000 h^−1^). Despite an overall yield of 99% in all the cases, the supported catalyst was slightly less active than the other two systems. The decline in activity, however, can be largely justified if either the separation costs from a homogeneous phase, or the production and disposal costs involved in a reaction conducted with a biphasic system are taken into consideration. In addition, the SILP system developed showed a negligible leaching (around 0.125 mol%), an effective parameter toward the reusability of the SILP catalyst.

## 6. Heterogenization of Iron Compounds on Chitosan

The progressive development of supports consisting of biodegradable, non-toxic, biocompatible, and available in large quantity biopolymers represents another answer to the problem of environmental sustainability [99]. Within this field lignin, collagen, wool, alginates, cellulose, and chitosan are among the most studied sources [100,101,102]. The latter is a derivative of chitin, a polysaccharide of *N*-acetyl-d-glucos-2-amine, held together by β(1-4)glycosidic bonds. Chitin, with its production of 1011 tons per year, is the second most abundant polysaccharide on Earth [103]. It is the main constituent of the exoskeleton of numerous animal species such as butterflies and ladybugs, but also shrimps, crabs, lobsters, and numerous other crustaceans. The food waste from the fish industry is today the main source of chitin and it is extracted through a process of decalcification, deproteination and removal of pigments [103,104,105].

**Figure 18:**
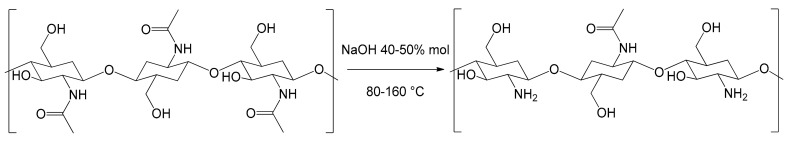
Synthesis of chitosan by partial deacetylation of chitin.

Chitosan is obtained from chitin by partial deacetylation of the polymeric chain by action of a strong base (Figure 18), leading to a copolymer of glucosamine and *N*-acetyl-d-glucosamine. The relative ratio of the two monomers defines the degree of deacetylation (DAD); properties such as viscosity, basicity and solubility depend on the DAD and average molecular weight. Chitosan possesses, by virtue of the large number of amino groups, extraordinary chelating abilities, particularly attractive for the immobilization of metal cations such as, for example, Fe^3+^. Furthermore, chitosan is soluble in an acidic medium due to protonation of the –NH_2_ groups, while it begins to precipitate at pH ≈ 6. Chitosan was initially used as a heterogeneous, basic catalyst in Michael’s addition reactions [106]. Unfortunately, it tends to gel in an aqueous environment due to its numerous hydrophilic groups, and this highlights a major limitation in the ability to recover and reuse the catalyst, and makes the separation of the products extremely complex [107].

**Figure 19:**
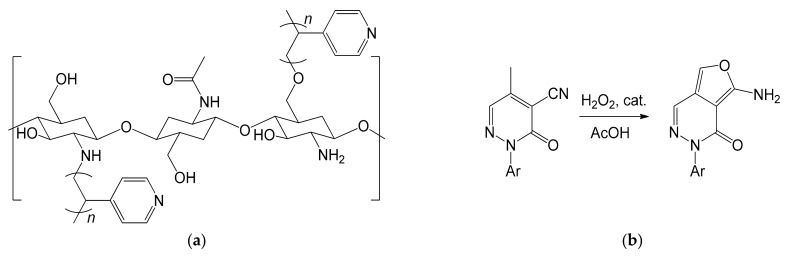
(**a**) Chitosan polymeric chain grafted with polyvinylpyridyl pendants; (**b**) Oxidation reaction of methylpyridazone using the grafted chitosan with immobilized Fe^3+^ ions as catalyst (Ar = phenyl, 4-chlorophenyl) [108].

In 2013, Al-Matar et al. published an effective method to overcome these drawbacks, managing to obtain a material less prone to gel, but equally effective in catalysis [108]. This objective was achieved by grafting the chitosan polymeric chain with polyvinylpyridyl pendants through suspension polymerization (Figure 19a). The powdered chitosan was poured into water and kept in suspension under stirring. The monomer 4-vinylpyridine was then added, together with a redox initiator, consisting of a 1:0.75 solution of K_2_SO_4_ and NaHSO_3_. The co-polymer was isolated after filtration and extraction of the unreacted monomer. The irregular particles were then re-dissolved in 5% (*v*/*v*) acetic acid to form a gel, which was sprayed onto a 0.50 M NaOH bath [108]. The strong base allowed them to neutralize the acid with consequent coagulation of the co-polymer into small but very porous spheres, thus increasing the surface area. The catalyst was therefore tested in Michael’s addition reactions, reporting acceptable results, and managing to easily separate the catalyst through simple filtration.

The real strength of the study was to exploit the ability of the obtained co-polymer to complex metal cations. Researchers hypothesized that the introduction of pyridine groups could have increased the ability to coordinate metal cations, supported by some publications on the subject [109,110]. This hypothesis was verified by introducing the spheres of grafted co-polymer in a 10 mM Fe^3+^ solution at pH = 1.8 for 5 h. The amount of immobilized iron ions was found to increase, going from 0.077 (mmol of Fe^3+^/g of chitosan) for the non-grafted chitosan to 0.086 (mmol of Fe^3+^/g of chitosan) of the grafted co-polymer. The obtained material was then successfully used in the oxidation of methylpyridazinone (Figure 19b) [108]. The reaction is of great interest, as the product obtained could be an important precursor for the synthesis of compounds such as phthalazine and its derivatives. The oxidation reaction took place by slow addition of H_2_O_2_ to a preheated solution of methylpyridazone in acetic acid. The whole reaction mixture was then heated under reflux in the presence of the heterogeneous catalyst. At the end of the reaction, the product was extracted using CH_2_Cl_2_. The catalyst, separated by filtration, was reused five times, without reporting significant drops in activity.

**Figure 20:**
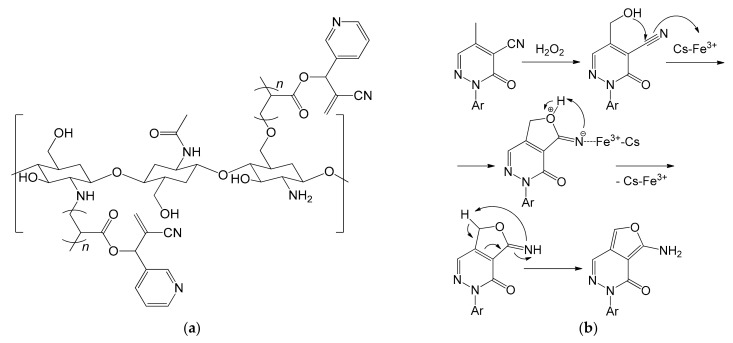
(**a**) Chitosan polymeric chain grafted with 2-cyano-1-(pyridin-3-yl)allyl acrylate (CPA); (**b**) Proposed mechanism of the oxidation reaction of methylpyridazone using grafted chitosan with immobilized Fe^3+^ ions (Cs–Fe^3+^) as catalyst (Ar = phenyl, 4-chlorophenyl) [111].

It is interesting to note how the same reaction was again the subject of another study a few years later [111]. Khalil et al. carried out the same oxidation reaction using Fe^3+^ ions immobilized in a chitosan biopolymer grafted with 2-cyano-1-(pyridine-3-yl)allyl acrylate (CPA) (Figure 20a). The methods used for the synthesis of the biopolymer, for the transformation into spheres and for the complexation of iron were the same as previously reported [108]. The support, however, showed a greater ability to immobilize the iron cations (0.091 mmol of Fe^3+^/g of chitosan) and the catalyst, overall, led to a higher reaction yield [111]. The authors also proposed a reaction mechanism consisting of four steps (Figure 20b). First, the methyl group is oxidized by the mixture H_2_O_2_/Fe^3+^, leading to the formation of a primary alcohol. This performs a nucleophilic addition on the adjacent –C≡N group with closure of the ring. The role of the catalyst is supposed to be crucial in this step since it should stabilize, through the Fe^3+^ cations, the nitrogen of the cyano group. A proton exchange between oxygen and nitrogen would then lead to the final product. The main difficulties that remained unsolved regarding the use of chitosan are substantially linked to its synthesis, since it requires the use of strong, dangerous and impacting acids and bases: 1 kg of chitosan with a DAD of 70% requires the use of 6.30 kg of HCl and 1.8 kg of NaOH [112]. Anyway, this could be overcome by resorting to the use of enzymes [103].

## 7. Conclusions

The investigation, discussed here, on the strategies adopted to heterogenize iron-based catalysts has brought out some interesting aspects that need to be highlighted. The first is that iron compounds, despite having a very wide application in homogeneous processes, currently have a very small number of applications in heterogeneous catalysis. This limitation makes merit of the difficulty to unify divergent requests, such as good activity of the catalyst while being recyclable. However, this unification is possible, and the cases here examined are good examples for this purpose. Secondly, it should be emphasized how they fit into different catalytic contexts and how this determines the effective possibility of implementation.

If we consider the heterogenized iron complexes employed in the synthesis of polyolefins, it must be observed that they compete on a panorama that offers numerous other solutions, especially based on titanium and zirconium [113,114]. The intricate synthetic processes described above remove this perspective to date. Therefore, the realization of an effective heterogenization and, at the same time, simple processes is still needed to make the strengths offered by iron worthwhile. In all the discussed examples, a decreasing in activity upon heterogenization is accompanied by a significant increasing in the molecular weight of PE. Mesoporous materials such as MCM-41 and MCM-48 demonstrated a high capacity of encapsulation of various iron complexes giving satisfactory results in the oxidation of several substrates such as alkenes and alkanes. The heterogenization on Merrifield resin is more interesting as it constitutes a viable and low-cost alternative for the synthesis of carbamates and *N*-substituted ureas. The use of SILP catalytic systems, still in its infancy as immobilizing solutions, is also promising in relation to industrial applications, paving the way for a novel concept of heterogenization that minimizes the leaching drawbacks favoring catalyst activity and selectivity. Nevertheless, the advantages offered by the high chemoselectivity must balance the costs and the inconvenience of still having an organic solvent in the reaction environment. Finally, functionalized chitosan can be used both as a basic catalyst and as a bio-based support, with certainly promising future advancements. In addition, the great availability at low cost of the raw material surely acts as a great stimulus for its wider use.

In conclusion, the knowledge on heterogenization of iron catalysts is still underdeveloped, although the research has been rather continuous since 2000. Thus, a strong expansion of knowledge in the field could be expected in the coming years, following the performance of novel iron homogeneous catalysts to develop sustainable catalytic processes which need high selectivity. The latter being the cases in which recovery and reuse of molecular catalysts by immobilization become essential for future industrial applications.

## Figures and Tables

**Table 1:** Results obtained by Gibson et al. in the ethylene polymerization in solution ^1^ [45].

Complex	Pre-Cat (μmol)	MAO (mmol/eq)	Activity (g/mmol h bar)	Yield (g)	*M*_W_	*M*_n_	*M*_W_/*M*_n_
**1**	0.5	0.5/1000	5340	26.9	611,000	64,000	9.5
**2**	0.6	0.6/1000	9340	56.5	242,000	9600	25.3
**3**	0.6	0.6/1000	20600	123.5	148,000	14,000	10.7
**4**	0.6	0.6/1000	3750	22.8	313,000	3000	105.1
**5**	6	1.2/200	305	18.2	132,000	3400	38.9
**6**	6	1.2/200	560	33.7	108,000	1900	57.3
**7**	6	1.2/200	340	20.3	230,000	3900	58.4
**8**	6	1.2/200	550	32.8	152,000	1800	83.5

^1^ isobutane solvent, 10 bar of ethylene, reaction time 1 h.

**Table 2:** Oxidation of styrene in homogeneous and heterogeneous systems with FePcS [55].

Catalyst	Reaction Time (h)	Conversion (%)	Selectivity (%)		Yield (%) Benzaldehyde	TOF ^1^
			Benzaldehyde	Benzoic Acid		
FePcS	6	39.3	18.7	47.3	7.3	19.7
FePcS	24	57.8	36.2	36.3	20.9	28.9
FePcS/NH_2_-MCM-41	6	16.7	19.1	0	3.2	8.3
FePcS/NH_2_-MCM-41	24	46.9	20.2	0	9.5	23.5
FePcS/NH_2_-MCM-48	6	21.9	23.9	0	5.2	10.9
FePcS/NH_2_-MCM-48	24	65.5	21.4	0	14.0	32.7

^1^ TOF = turnover frequency expressed as mol styrene converted/mol Fe.

**Table 3:** Results of the catalytic hydrogenation reaction ^1^ of aldehydes in the presence of [Fe(PNP-Me-*i*Pr)(CO)(H)(Br)] tested by Kirchner et al. [93].

**Substrate**	**S/C ^2^**	**Conversion (%)**	**Yield (%)**	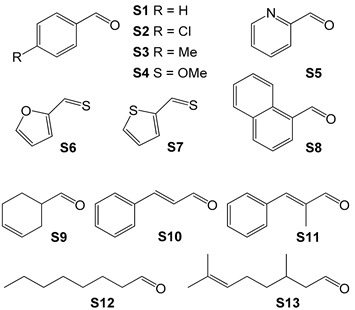
**S1**	20,000	>99	96	
**S2**	20,000	>99	>99	
**S3**	15,000	>99	>99	
**S4**	15,000	98	98	
**S5**	20,000	>99	97	
**S6**	20,000	>99	>99	
**S7**	20,000	>99	>99	
**S8**	10,000	97	96	
**S9**	10,000	>99	98	
**S10**	10,000	>99	>99	
**S11**	20,000	>99	>99	
**S12**	10,000	>99	>99	
**S13**	10,000	99	97	

^1^ reaction conditions: catalyst 0.1–0.2 μmol, aldehyde 2 mmol, DBU 1 mol%, EtOH 1 mL, 30 bar H_2_; 40 °C; 16 h; ^2^ substrate over catalyst ratio.

**Table 4:** Results obtained by Kirchner and collaborators from the hydrogenation ^1^ of 4-fluorobenzaldehyde using different SILP catalytic systems [94].

SILP System	H_2_ (atm)	S/C ^2^	*t* (min)	Yield (%)	TON	TOF (h^−1^)
SILP10	10	200	75	16	32	26
SILP20	10	200	17	>99	200	706
SILP30	10	200	75	7	14	11
SILP40	10	200	75	3	6	5
SILP20	20	200	13	>99	200	923
SILP20	50	200	8	>99	200	1500
SILP20	10	1000	90	85	850	567
SILP20	50	1000	15	>99	1000	4000
homogeneous	10	200	6	>99	200	2000
biphasic	10	200	12	>99	200	1000

^1^ reaction conditions: DBU (5 mol%), *n*-heptane, 25 °C; ^2^ substrate over catalyst ratio.

## Abbreviations

IL	Ionic Liquid
SILP	Supported Ionic Liquid Phase
MAO	Methylaluminoxane
MMAO	Modified MAO
PE	Polyethylene
LDA	Diisopropylamide
PS	Polystyrene
AIBN	Azobisisobutyronitrile
TOF	turnover frequency
TPP	Tetraphenylporphyrin
DBU	1,5-diazabiciclo(5.4.0)undec-7-ene
DAD	Degree of deacetylation
CPA	2-cyano-1-(pyridine-3-yl)allyl acrylate
^iPr^DPI	2,6-bis(2,6-diisopropylphenylimino)pyridine)
THF	Tetrahydrofuran
EtOH	Ethanol
DMF	Dimethylformammide
MeLi	Methyllithium
AcOH	Acetic acid
Et_2_O	Diethyl ether
MeOH	Methanol
PNP	Pincer ligand with phosphorus, nitrogen, phosphorus donor atoms
NNN	Pincer ligand with three nitrogen donor atoms
DFT	Density Functional Theory
bm2im	1-butyl-2,3-dimethylimidazolium
NTf_2_	Bis(trifluoromethylsulfonyl)imide
MCM-xx	Mobil Composition of Matter n° xx.
FePcS	Iron tetrasulfophtalocyanine
CPTES	3-chloropropyltrietoxysilane
MAS-NMR	Magic Angle Spinning-Nuclear Magnetic Resonance
en	Ethylenediamine
bpy	2,2-bipyridine
salen	*N,N*’-bis(salicylidene)ethylenediamine
TMC	5,7,12,14-tetramethyl-1,4,8,11-tetraazacyclotetradeca-4,6,11,13-tetraene
